# Ethical issues associated with medical tourism in Africa

**DOI:** 10.1080/20016689.2017.1309770

**Published:** 2017-05-05

**Authors:** John J. O. Mogaka, Lucia Mupara, Joyce M Tsoka-Gwegweni

**Affiliations:** ^a^ Discipline of Public Health Medicine, University of KwaZulu Natal, Durban, South Africa

**Keywords:** Health ethics, Africa, medical tourism, healthcare delivery

## Abstract

Global disparities in medical technologies, laws, economic inequities, and social–cultural differences drive medical tourism (MT), the practice of travelling to consume healthcare that is either too delayed, unavailable, unaffordable or legally proscribed at home. Africa is simultaneously a source and destination for MT. MT however, presents a new and challenging health ethics frontier, being largely unregulated and characterized by policy contradictions, minority discrimination and conflict of interest among role-players. This article assesses the level of knowledge of MT and its associated ethical issues in Africa; it also identifies critical research gaps on the subject in the region. Exploratory design guided by Arksey and O’Malley’s (2005) framework was used. Key search terms and prior determined exclusion/inclusion criteria were used to identify relevant literature sources. Fifty-seven articles met the inclusion criteria. Distributive justice, healthcare resource allocation, experimental treatments and organ transplant were the most common ethical issues of medical tourism in Africa. The dearth of robust engagement of MT and healthcare ethics, as identified through this review, calls for more rigorous research on this subject. Although the bulk of the medical tourism industry is driven by global legal disparities based on ethical considerations, little attention has been given to this subject.

## Introduction

### Case scenarios

There are people desperate for, and eager to pay for, organs of a brain-dead patient: should the doctors withhold all cardiopulmonary resuscitation (CPR) or advanced cardiac life support (ACLS) lifesaving efforts including the use of powerful drugs to keep the heart beating and manage blood pressure, or should they let him die fast for the sake of the potential organ recipients? Should the high cost of accessing gene therapy be the basis of making it available only to the able-to-pay? And should people be allowed to use it to enhance basic human traits such as height or athletic ability? Or, in the face of finite healthcare resources, should patients needing elective medical treatments receive equal attention as patients with life-threatening medical needs?

These are some of the healthcare ethical issues that medical tourism grapples with, especially in resource-limited settings of Africa.

Disparity in laws across the globe, emerging breakthrough medical technologies, global economic inequities and globalization synergistically promote medical tourism (MT) [1]. MT provides a platform for some patients to travel abroad for medical care that is either too delayed, unavailable, unaffordable or legally proscribed in their home countries [2,3]. Some of these patients leave Africa, others come into Africa, while others travel within the continent (intra-Africa) for various medical care services [4,5].

Travel motivations for patients vary. From outside Africa, long waiting-lists for certain procedures in some countries, and medical under- or non-insurance are motivations for travel, mostly from developed countries [6]. Lack of good quality hospitals and health professionals in most African countries cause some patients to travel internationally or regionally to other African countries, like South Africa, Tunisia, Egypt and Mauritius for medical care [4,7]. Some countries adopt restrictive regulations on certain treatments, such as some stem cell therapies, surrogate pregnancy, organ transplantation and sex determination for the unborn, thereby causing patients who want or need these treatments to travel to where they are either legal, unregulated or less restricted [1,2].

Largely, MT packages medical care as a tradable commodity and avails it in the global healthcare market, mainly using tourism channels. As the global MT platform expands, however, auxiliary healthcare players have emerged, acting as promoters or brokers between international patients and healthcare providers [8]. Even though these ‘meso-cadre’ healthcare professionals help arrange surgeries, travel arrangements and recuperative tours, their training, affiliation and professional commitment is not succinct. Nonetheless, besides mere provision of, and facilitation for, affordable accommodation and wide ranging logistical services for medical care, these players create opportunities for people to access exceptional medical treatments while enjoying tailored luxury vacations in the process.

MT makes many treatments that used to be affordable only for the elites in society readily accessible and available at a number of destinations around the world, including Africa [9,10]. Cutting-edge medical treatments are made candidly accessible through MT, offering many patients a chance to undergo unique procedures they could otherwise not access at home.

Many developing countries link medical care with tourism, and aim at maximizing benefits from the resulting fiscal transactions to further develop their economies [9,11–13] . Although medical treatments and procedures are becoming commodities which can be sought, bought, traded and sold, [14] and can improve individual patient experiences in terms of treatment outcomes, MT raises ethical issues, both at the personal and population health levels.

Ethical issues in MT cross-cut the more clinical, bedside biomedical ethics, bioethics and the wider healthcare ethics, as MT encompasses concerns faced by health professionals, health policy-makers, patients, families and communities in wide-ranging healthcare settings, including patient care, healthcare delivery in national health systems, global public health and medical technologies. Healthcare is essentially a moral enterprise [15]; a fact that necessarily demands a thorough assessment of ethical issues associated with MT.

Despite a substantial lack of empirical research on the effects of MT on health ethics in Africa, many researchers have, however, noted several ethical and public health concerns relating to MT, particularly its potential impact at both individual and societal levels within destination and source (departure) countries [1,6,16–28].

## Research objectives

To date there is no known survey carried out on MT and associated health ethics in Africa, despite the fact that ethical issues influence policy decisions on, and hence legal status of, some medical procedures and treatments. This study aimed at establishing the level of knowledge of ethical concerns of MT in Africa. Specific objectives of this synthesis report were:

1. to synthesize existing knowledge on MT in Africa as it relates to ethics and public health;
2. to identify what is and is not known about MT and health ethics; and uncover patterns of findings in the field;
3. to identify areas of controversy and consensus in the literature and identify knowledge gaps that need further research on the subject.

This article, however, is not an exhaustive description of actual and potential ethical concerns of MT in Africa; nor does it facade a single correct stance; rather, it examines the variation and complexity of different theoretical conceptualizations and engages with the debates that have taken place on the topic, mostly from epistemological perspectives.

To achieve these objectives, the following methodology was applied.

## Methodology

The present study adopted the scoping review method because it aimed at identifying research and information gaps that exist regarding ethical issues associated with MT as an emerging subject of interest [29–32]. The review followed Arksey and O’Malley’s methodological framework for scoping reviews [29,30].

In this scoping review, methodological quality assessment of quantitative, qualitative and mixed methods primary studies was done on relevant admitted studies using the Mixed Methods Appraisal Tool (MMAT) [33]. However, this assessment was not done to exclude studies on account of quality scores; rather, quality scores were considered in the narrative synthesis of the evidence.

The design followed a five-step process of: identification of the questions to be addressed; identification of the relevant literature sources; selection of literature sources to be included in the present review synthesis; recording key themes emerging from the literature; and collation, summary and reporting of the results [32].

### Inclusion and exclusion criteria

**Figure 1.**
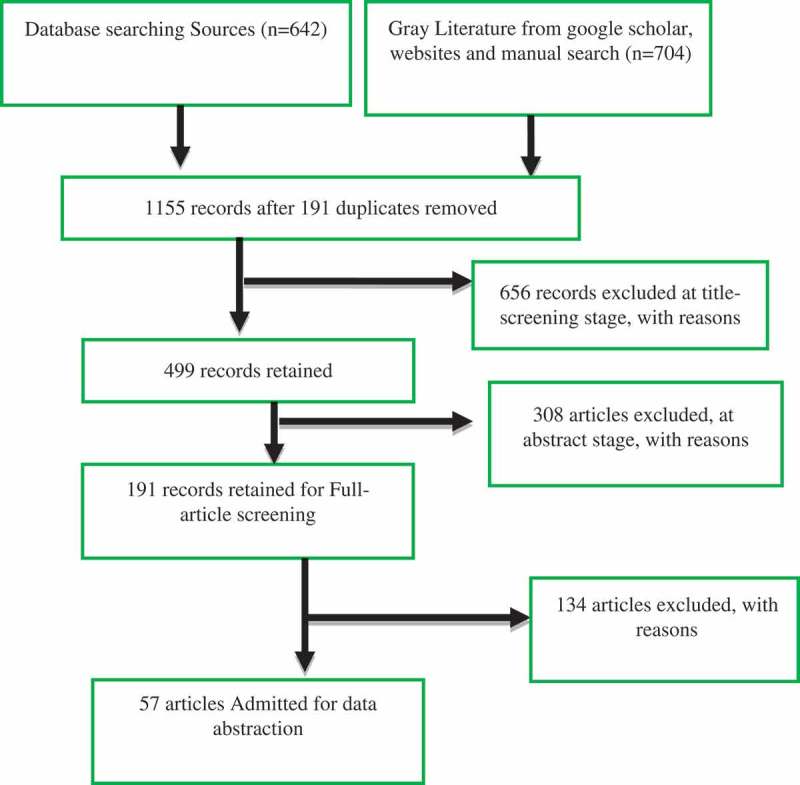
PRISMA record screening flowchart. (Source: Adapted from Moher et al. [34]).

Inclusion/exclusion criteria based on the review objectives were devised and refined during the first stage of selection for literature retrieval. Figure 1 shows the search results after applying the pre-determined inclusion/exclusion criteria using the Preferred Reporting Items for Systematic Reviews and Meta-Analyses (PRISMA) Record Screening Flow-chart, adapted from Moher et al. [34].

Two researchers independently read the first 30% of abstracts and decided whether the inclusion criteria applied. Their decisions were compared, and a kappa index [35] calculated. The score was again calculated at the end of full article inclusion stage. Disagreements were discussed among the reviewers and differences in interpretation were clarified. All papers meeting the inclusion criteria at this stage were selected for retrieval.

Those studies that were judged by agreement to address the review topics sufficiently were retained.

### Sources of literature

The sources of information used included databases: Regional Business News; PsycINFO; MasterFILE Premier; Health Source: Nursing/Academic Edition; GreenFILE; ERIC; Education Source; Business Source; Ultimate Business Source Complete; Newspaper Source; Library, Information Science & Technology Abstracts; Health Source – Consumer Edition; eBook Collection (EBSCOhost); AHFS Consumer Medication Information; PsycARTICLES; MEDLINE with Full Text; Academic Search Ultimate; and Academic Search Complete (n = 18).

A Google scholar search was performed to identify relevant gray literature, which included unpublished conference papers and abstracts, government websites, books and news articles. The websites of key medical tourism organizations and associations were also searched.

The team collected potentially relevant citations from reference lists and applied the refined inclusion criteria on them.

Data on the study setting and the key findings described in each article were recorded and organized into different themes in NVIvo. Information obtained included the place where the research was conducted (e.g., low- and middle-income countries [LMIC]/high-income countries [HIC]), the type of study (e.g., empirical, review, expert opinion), the type of analysis techniques used (e.g., statistical analysis, thematic analysis) and findings applicability (Africa local/regional or global).

## Results

Our search identified 1,346 potentially relevant articles in the scoping review. Using endnote reference management software, duplicate studies were removed. The remaining 1,155 were screened for title relevance. Four hundred and ninety-nine articles underwent a detailed abstract screening against inclusion criteria. One hundred and ninety-one articles were selected for full-article screening by two researchers, with 57 being selected for independent detailed (full) data abstraction for this synthesis. They were also included for methodological quality assessment.

The inter-reviewer kappa score was 0.89 at abstract screening stage and 0.83 at full article screening stage.

Of the 499 included papers at abstract screening stage, 308 were excluded, as shown in Table 1.

**Table 1.** Criteria for excluding papers at abstract screening stage (n = 308). (Source: Authors).

No of records excluded	% of total included (^n^/_499_)	Reasons for exclusion
169	34	Medical care provision to medical tourists is not explicitly differentiated from the day to day provision of health care offered to the general public
61	12	Main focus is on wellness tourism
78	15	Focus on MT outside Africa and results/conclusions are non-transferable to African settings

Of the 191 articles assessed for eligibility for full screening, ^19/^_191_ (less than 10%) articles focused specifically on MT in Africa [4,5,7,13,27,28,36–48].

**Figure 2.**
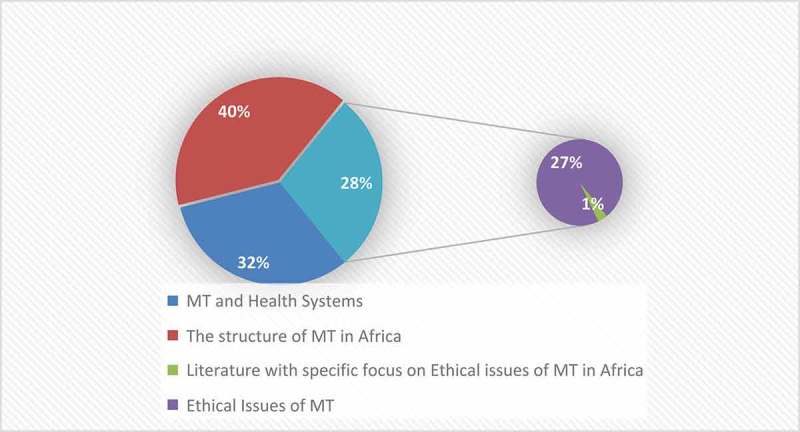
Themes explored on MT in Africa. (Source: Authors).

**Figure 3.**
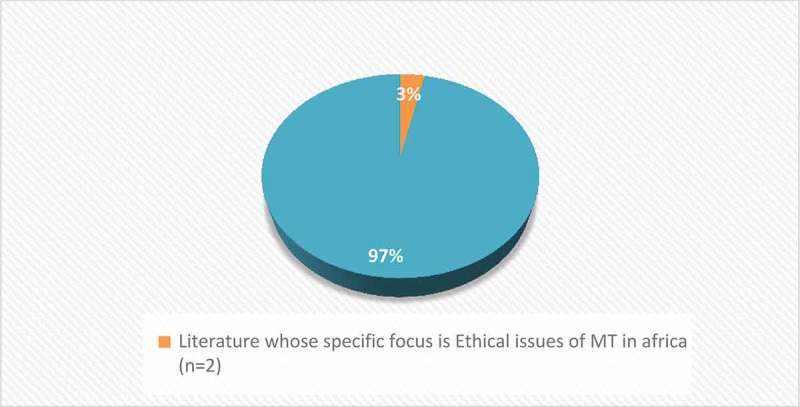
Research with exclusive focus on MT ethical issues in Africa (n = 2) compared to total research on ethical issues of MT globally (n = 57).

**Figure 4.**
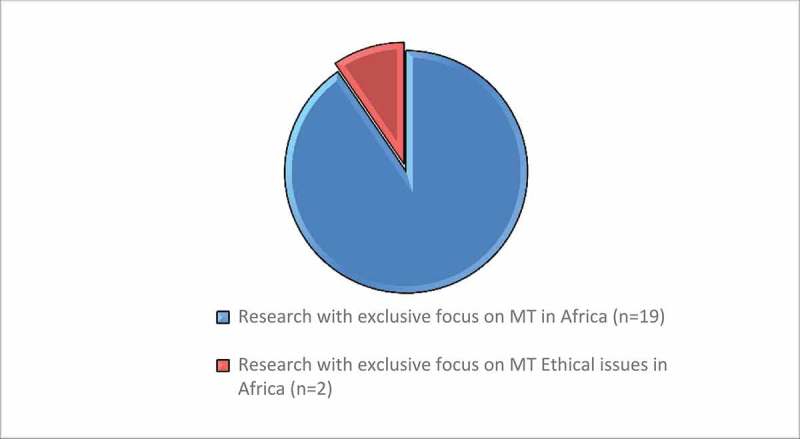
Research with exclusive focus on MT ethical issues in Africa (n = 2) compared to total research on MT in Africa(n = 19).

Of the 19 articles, only ^2^/_19_ [27,46] dwelt on ethical issues of MT in Africa. These two sources focused on stem cell and surrogacy tourism (reproductive tourism) respectively. Figures 2–4 show the attention given to ethical issues of MT in Africa at various levels. Figure 2 shows that of the 191 articles assessed for admission eligibility in this study (n = 191), only 57 (28%) focused exclusively on ethics of MT globally, out of which only 1% were focusing exclusively on ethical issues of MT in Africa.

**Figure 5.**
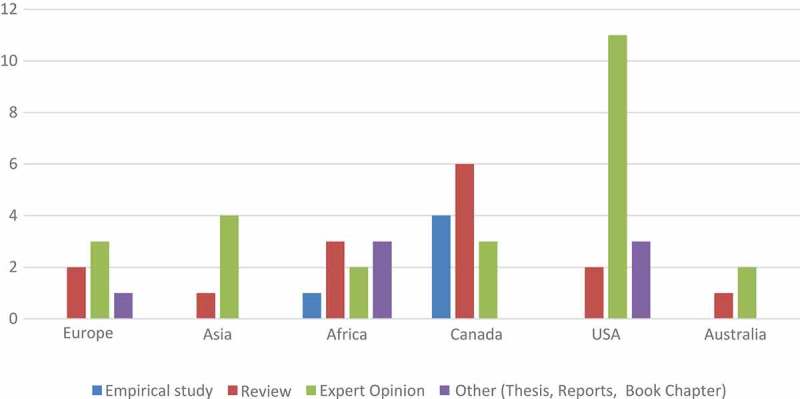
Type of papers identifying ethical issues of MT in Africa and author location. (Source: Authors).

Figure 5 shows the nature of the papers of the included sources that focused on ethical issues of MT in Africa, either specifically or generally, as compared to authors’ geographic location.

**Figure 6.**
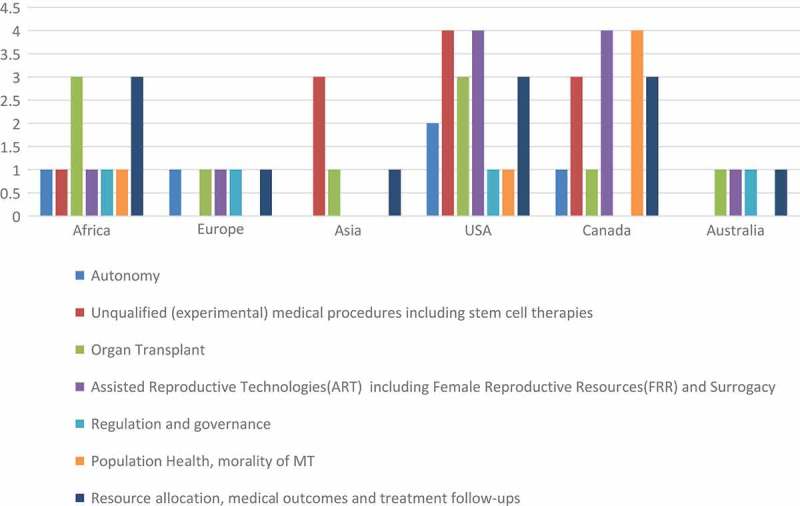
Main ethical issues identified in literature applicable to MT in Africa and location of the identifying source. (Source: Authors).

Figure 6 shows the main ethical issues identified in the literature whose focus is either specifically on MT in Africa or ethical issues of MT globally but applicable to the African settings.

## Discussion

The methodological suitability of systematic scoping review employed in this study was informed by the fact that, unlike the traditional systematic reviews, scoping reviews are characterized by breadth of coverage and ability to include a wide range of publications and study designs [29,30,49–51], to particularly help in mapping relevant literature.

### Methodological aspects of the reviewed literature

The papers included in this study have diverse methodologies, individually employing a range of study designs, paradigms and report formats for various purposes. For that reason, the evidence was subjected to methodological screening, in an attempt to sum up the range of research methods used and discuss arising methodological challenges and issues.

As shown in Figure 5, most ethical issues on MT identified for Africa are actually generalized global MT ethical issues as reflected in papers with a global MT outlook. Evidently, most of these papers originate from North America (Canada and USA), Asia and Europe, with the majority being either reviews or expert opinion articles (Figure 5). Although few in number, almost all the empirical research studies on MT and healthcare ethics are qualitative. This study found the overall paucity of rigorous empirical studies on ethical issues of MT in Africa a factor that severely undermines the basis for the many reviews and expert opinion reports that feature prominently in this field. Notably, the few empirical studies conducted in Africa are medical case based [37,44], secondary-data and legal case reports[4,5]. For instance, one research had ‘informed observers’ interviewees who were asked of their experience with MT ethical issues in developing countries (LMICs) [16], whereas the entire set of study participants in the study is based in a developed country (i.e., Canada). This begs the question: if ethics is concerned with the values underlying decisions and actions, what values and whose values are relevant?

This scenario immediately suggests two important epistemological implications for such qualitative studies:

1. Due to the fact that positionality plays out in most qualitative research and expert opinion reports [52], an investigator’s placement within the many contexts, layers, power structures, identities and viewpoints particularly influences his/her conclusions, and implications from the findings of such a qualitative study inquiry are particularly subjective [52,53].
2. In attempts to generalize some of the global MT ethical issues, there is potential to inappropriately exclude the voices of the local people, in this case Africa, thereby, albeit unintentionally, reinforcing patterns of North–South domination.

On the other hand, however, due to contextual realities, qualitative studies tend to include much more contextual data, which facilitates more informed judgments about phenomenal dynamics of ethical issues in MT. Also, it is not always that ethical theories and principles can be applied on their own to address all ethical uncertainties for patient groups, communities and populations. Therefore, expert opinion is often a valuable resource to inform such decisions.

The following methodological challenges and issues in MT research have been noted in this study:

1. Empirical reality in MT research is complicated. There is a lack of reliable, internationally comparable data, including basic information on the volume and value of the trade through MT, basically arising from lack of consensus on the concept, and data standardization of MT globally [54,55]. Most health systems have different accountability frameworks at different institutional levels. Thus, isolating and rigorously exploring the ethical issues of MT is compounded by this empirical reality.
2. MT as a research field is relatively new [54]. The contours of this phenomenon are just beginning to emerge. But most poignantly, the difficulty in getting access to international patients, and patients’ reluctance to participate in research, are particular complication factors in this field [56].

## Identified ethical issues of MT in Africa

The main aim of this report was to synthesize existing knowledge on MT in Africa as it relates to ethics in healthcare by identifying what is and is not known and areas of controversy and consensus in the literature. However, the reviewed works point to an apparent lack of reconcilable consensus on the moral concepts of ethical issues associated with MT.

Major areas of discourse and debate in extant literature include:

- the belief that healthcare, specifically medical care through investment in public health, should produce health benefits for all in society, reflected in utilitarian health policies;
- respect for individual autonomy and liberty of action for healthcare seekers;
- protection and promotion of minority group healthcare needs by avoiding discrimination, marginalization and stigmatization;
- distributive and procedural justice, fair distribution of healthcare, ensuring accessibility, participation and accountability.

In the reviewed literature, this study observed an entwinement of the theoretical and empirical in the discourses and debates on ethical issues of MT. Empiricism here means the experiential and/or practical instances where issues were identified and deemed to be MT ethical issues based on the proficiency and judgement of various researchers and/or authors. On the other hand, the theoretical is taken to mean the deliberations based on moral theories to advise how moral agents ought to act and the process of isolating ethical concepts and the nature of their justification using philosophical reasoning. Due to the evident empirical–theoretical intertwinement, the boundary between philosophical reasoning and empirical research conclusions on ethical issues associated with MT in Africa is unclear. Furthermore, sifting through the reviewed literature revealed that most of the work did not directly point out ethical issues associated with MT; instead, inference of these issues had to be assumed. Consequently, answers to the quest to gain knowledge on the identified ethical issues associated with MT in Africa were mined from sources severely encumbered with numerous other factors e.g., theories, assumptions and study settings. This further buttressed the necessity to juxtaposition empiricism and normative ethics to best provide a more thorough understanding of the many perspectives presented in the reviewed works. Therefore, while recognizing the identified ethical issues in the reviewed works, this article goes beyond the surface to critique the epistemological underpinnings of these ethical issues, in an attempt to uncover any research gaps with special reference to limited resource settings of Africa. Therefore, this article assumes a critical discussion and analysis outlook.

MT is driven by social, legal, technological and economic disparities at regional and global levels. Efforts to improve the health of some populations sometimes unintentionally makes the other population worse off, raising ethical conflicts in deciding ‘right’ and ‘good’ actions, both perceived and real.

From the reviewed works, the identified ethical issues generally correspond to three ethical theories based on

- the consequences of an act (consequentialism);
- the agent carrying out the act (virtue ethics theory);
- the act itself (deontology).

Each theory differs from the other in content and application, such as predicting the outcome and following one’s duties to reach an ethically correct decision. However, as shown in Table 2 and Figure 6, most reviewed works have avoided basing their arguments on definitive moral theories in favour of various modes of moral reasoning falling on a spectrum continuum between casuistry or case-specific ethical decisions, on one hand, and the mid-level bioethical norms of principlism, on the other. Bioethical principles, derived mostly from the Hippocratic Oath and Beauchamp and Childress (1994)’s four ethical principles, feature prominently in the reviewed literature. The principles include:

- beneficence;
- non-maleficence;
- respect for persons; and
- justice.

**Table 2.** Data abstraction results.

Author, year	Country/region	Study setting	Main ethical issues	Exclusive on MT ethics?	Design/methodological approach	Main focus is on MT ethics in Africa?
Adams, Krystyna; Snyder, Jeremy Crooks, Valorie A.	Canada	HIC Global	Information given to medical tourists		Qualitative	No
Johnston, Rory (2013)						
Bagheri, Alireza (2010)	Iran, Asia	LMIC, Global	Organ transplant	Yes	Expert opinion	No
Barclay, Eliza (2009)		Global	Unproven stem cell therapies	Yes	Expert opinion	No
Bezabih, B.	Ethiopia, Africa	Africa, local	Cultural, economic and quality issues	No	Medical records, review	No
Wamisho, B. L. (2013)						
Chou, Franklin (2016)	USA	HIC Global	Volume shock – causing the failure of both global and domestic health care frameworks	No	Thesis	No
Cohen, I. Glenn (2010)	USA	HIC Global	Illegal medical procedure, resource allocation	Yes	Expert opinion	No
Cohen, I. Glenn (2011)	USA	HIC Global	Resource allocation. Access	Yes	Expert opinion	No
Cohen, I. Glenn (2012)	USA	HIC Global	Regulatory issues	Yes	Expert opinion	No
Cohen, I. Glenn (2013)	USA	HIC Global	Kidney transplant ethical issues	Yes	Expert opinion	No
Connell, John (2011)	Australia	LMIC, Global	Resource allocation. Access	Yes	Expert opinion	No
Crooks, Valorie A.	Canada	LMIC, Local	Resource allocation. Access	No	Empirical qualitative	No
et al. (2015)						
Crooks, Valorie A.et al. (2010)	Canada	HIC Global	Resource allocation, Commodification of healthcare	No	Scoping review	No
Crozier, G. K. D.	Canada	HIC Global	Trade in female reproductive resources: ova and surrogacy	Yes	Expert opinion	No
Martin, Dominique (2012)						
Crozier, G. K. D.	USA	HIC Global	Minors’ stem cell therapy	Yes	Expert opinion	No
Thomsen, Kyle (2010)						
Crush, Jonathan	South Africa, Africa	Africa, regional	Medical exclusion	No	Secondary data sources	No
Chikanda, Abel (2015)						
Crush, Jonathan	South Africa, Africa	Africa, regional	Global trafficking of illegal organs for transplant	No	Secondary data sources	No
Chikanda, Abel						
Maswikwa, Belinda (2012)						
Crush, J. et al. (2013)	South Africa, Africa	Africa, regional	Global trafficking of illegal organs for transplant	No	Secondary data sources	No
Dolan, Timothy (2010)	S. Korea, Asia	LMIC, Global	Stem cell therapy	Yes	Expert opinion	No
Einsiedel, Edna F.	Canada	HIC Global	Stem cell therapy	Yes	Empirical qualitative	No
Adamson, Hannah (2012)						
English, Veronica et al. (2005)	USA	HIC Global	Right to require treatment	Yes	Review	No
Hadi, Abdullahel (2009)		HIC Global	Commodification of healthcare	No	Conference paper	No
Harrison, Laura (2014)	USA	HIC Global	Cross racial gestational surrogacy	Yes	Expert opinion	No
Hede, Karyn (2012)	USA	HIC Global	Stem cell therapy	Yes	Expert opinion	No
Honey, Karen (2009)		Global	Organ transplant	Yes	Expert opinion	No
Hunter, David	USA	Global	Illegal medical procedure issues	Yes	Expert opinion	No
Oultram, Stuart (2010)						
Idowu, Emmanuel Olufemi	Nigeria, Africa	Africa, local	Medical outcome disenfranchized medical tourists	No	Empirical, medical records	No
Adewole, Oladipo Adeboluji						
Inhorn, Marcia C.	USA	HIC Global	ARTs	No	Expert opinion	No
Patrizio, Pasquale (2009)						
Levine, Aaron D.	USA	HIC Global	Stem cell therapy	Yes	Expert opinion	No
Wolf, Leslie E. (2012)						
Lozanski, Kristin (2015)	Canada	HIC Global	Transnational surrogacy	Yes	Expert opinion	No
Lunt, Neil	Europe	HIC Global	Follow-up care; proscribed medical procedures	No	Review	No
Carrera, Percivil (2010						
Manzano, Ana et al. (2014)	UK, Europe	HIC Global	Organ transplant	Yes	Review	No
Martin, Dominique E. (2016)	Australia	HIC Global	Kidney transplant ethical issues	Yes	Expert opinion	No
McMahon, Dominique	Canada	HIC Global	Stem cell therapy	Yes	Expert opinion	No
Thorsteinsdottir, Halla (2010)						
Meghani, Zahra (2011)	USA	HIC Global	The morality of MT	Yes	Expert opinion	No
Meissner-Roloff, Madelein	South Africa, Africa	Africa, regional	Stem cell therapy	Yes	Expert opinion	Yes
Pepper, Michael S. (2013)						
Mutalib, Nur Syafiqah Abd et al.	Malaysia Asia	LMIC Global	Resource allocation issues	No	Review	No
(2016)						
Nicolaides, A.	South Africa, Africa	Africa, regional	Organ transplant	Yes	Review	No
Smith, A. (2012)						
Osland, Asbjorn	USA	LMIC Global	Surrogacy	Yes	Critical incident	No
Clinch, Nanette (2013)						
Panitch, Vida (2013)		Global	Surrogacy	Yes	Review	No
Patoine, Brenda (2008)	USA	HIC Global	Stem cell therapy	Yes	Review	No
Pfeffer, Naomi (2011)	Europe	HIC Global	Female reproductive resources	Yes	Expert opinion	No
Shalev, Carmel (2010)	Israel, Asia	LMIC Global	Stem cell therapy	No	Expert opinion	No
Sipp, Douglas (2010)	Japan Asia	HIC Global	Stem cell therapy	No	Expert opinion	No
Sixty-Third, World Health Assembly (2010)	Global	Global	Organ transplant	No	WHO report	No
Skountridaki, Lila (2015)	Europe	Global	Conflict of interest in medics as MT entrepreneurs	No	Expert opinion	No
Smith, Elise et al. (2010)	Canada	HIC Global	Reproductive tourism	Yes	Review	No
Smith, Kristen (2012)	Australia	HIC Global	Conflicting link between government and industry in MT	No	Review	No
Snyder, Jeremy et al. (2013)	Canada	HIC Global	Responsibility for MT harms	No	Review	No
Snyder, Jeremy et al. (2011)	Canada	HIC Global	Individual medical and population health ethics	Yes	Review	No
Snyder, Jeremy	Canada	HIC Global	Medical follow-up; Resource allocation	Yes	Review	No
Crooks, Valorie A. (2010)						
Snyder, Jeremy	Canada	HIC Global	Experimental and reproductive treatments; organ transplantation	Yes	Review	No
Crooks, Valorie A. (2012)						
Umeora, Odidika Ugochukwu et al. (2014)	Nigeria, Africa	Africa, regional	Surrogacy	Yes	Review	Yes
Uppiah, MV et al. (2014)	Mauritius, Africa	Afrca, local	MT legislation	No	Expert opinion	No
Voigt, Cornelia	Australia	HIC Global	Commodification of reproduction	Yes	Review	No
Laing, Jennifer H. (2010)						
Whitmore, Rebecca et al. (2015)	Canada	HIC Global	Ethics of care in MT	Yes	Empirical	No
Widdows, Heather (2011	Europe	HIC Global	Inadequacy of informed consent in medical tourism and population genetics.	Yes	Expert opinion	No

MT encompasses a variety of distinct but interrelated activities, some of which might be more amenable to some philosophical theories than others. From the reviewed works, three levels of health ethics can be deduced:

- Biomedical ethics at the most basic, immediate, clinician-patient contact level. The clinical bioethics involve physicians, nurses, social workers, patients or their family members who ask for assistance in resolving actual clinical cases, in real time.
- Policy-oriented bioethics. Here, bioethics informing policies that affect large numbers of people are formulated at the level of individual healthcare institutions or facilities such as hospitals and national health systems. Discussions often focus on the merits of competing policies such as quantitative and qualitative medical futility or do-not-resuscitate orders; or they can take place in the atmosphere with various national health systems charged with formulating policy on topics such as cloning, healthcare resource allocation, organ transplantation, or even assisted suicide.
- Theoretical ethics development at academic and research level, which is unhindered by time constraints, medical custom, law, or the need for a timely decision, but aiming at furthering healthcare ethics realms.

Table 3 summarizes the ethical theories, principles and the most commonly observed ethical issues associated with MT in Africa, as identified in the reviewed literature.

**Table 3.** Identified ethical issues of MT in Africa and corresponding ethical theories and principles. (Source: Authors).

		Identified MT in Africa ethical issue						
Type of ethical theory		MT and population health.	Morality of MT	Resource allocation, including medical outcomes and treatment follow-ups.	Assisted reproductive technologies(ART) including female reproductive resources (FRR) and surrogacy	Organ transplant	Unqualified (experimental) medical procedures including some stem cell therapies	Freedom for patients to choose
Consequence-based (act and rule utilitarian)		√√	x	√√	√√	√√	x	√√
Duty-based (deontology)		√√	x	√√	x	√√	x	x
Pri-	Beneficence	x	x	x	√√	√√	√√	x
ncip-	Non-maleficence	x	x	x	√√	√√	√√	√√
lism	Autonomy (respect for persons)	x	x	√√	√√	√√	√√	√√
	Justice	√√	x	√√	√√	√√	√√	x
Character-based (virtue)		x	x	√√	x	x	√√	x
Contract-based (rights)		√√	x	√√	x	x	x	√√

Some reviewed works oppose MT in developing countries. Basing their arguments on deontological ethical theories, emphasizing that people should adhere to their obligations and duties to society. They argue that MT is a neo-colonialism product which has caused a two-tier healthcare system in the developing countries [57,58]. This dichotomy is especially pronounced in developing countries, with one tier providing ‘excellent treatment in technologically sophisticated modern hospitals catering to foreigners and local elites, whilst, despite their many and pressing problems, large sections of the rest of the population are unable to access or afford the basic health care provided at a price by the other tier’ [59]. Neocolonialism tenets of lowering barriers to global trade, promoting markets, privatizing public services, including health, and pursuing small government and encouraging governments to eliminate subsidized or free basic health care for local populations are blamed for this outcome. Proponents of MT, however, embrace teleological theories of utilitarianism and libertarianism which emphasize freedom, individual liberty and voluntary association. They project MT as a platform that gives people freedom to choose health providers, and to access care that is either too delayed, unavailable, unaffordable or legally proscribed in their home countries [1,2]. Some question ethical assumptions and claims that some intimate human functions and experiences, such as pregnancy and childbirth are intrinsically unsuitable for sale, and so should not be taken to the market [60].

Whether MT is moral or not needs an engagement with the claims, assumptions and critiques made at a meta-ethical theoretical level as argued in some reviewed works [61].

Based on ethical principlism of autonomy, informed consent and veracity, some reviewed works have pin-pointed how MT ‘packages’ are promoted to international patients by destination hospitals, companies and government agencies [23] particularly as problematic. This is regarding the ethical principle that instructs practitioners to be honest in their dealings with patients and give full disclosure of risks and benefits of treatments. These arguments, however, employ more casuistry and narrative ethics, eschewing the more philosophical ethical theories.

The character-based virtue ethical theory places emphasis on the value of autonomy above other ethical values in the physician–patient relationship, particularly promoting medical paternalistism. The physician’s character is regarded beyond censure, the belief that the physician knows best, with any advice from the physician for an operation, laboratory test or medication taken without much interrogation. But the principle of autonomy, especially the sufficiency of informed consent, in MT has been critiqued and found particularly unsatisfactory [1].

Through MT, and based on libertarian and other rights-based ethical theories, patients’ wishes have been prioritized over their best interests. This places patients in much control of their own healthcare decisions, though without much reliable information, leaving him/her to live with the consequences and quality of life so chosen. This results in ethical issues associated with follow-up treatments, whether abroad or at home [62]. Although MT places more emphasis on the patient’s desires, it does not prevent the patient from making decisions that may be more harmful than beneficial. Here, the patient is turned into a medical client and true healthcare consumer, based on the ability to pay. Nowhere is this more pronounced than in transplant and surrogacy tourism. Here, the physician’s fidelity obligations are at odds. The organ recipient and the commissioning parents, the parties that are paying for the services gets the weightier covenantal fidelity while the organ sellers and surrogate mothers get the lesser contractual fidelity, with resulting ethical intricacies.

The concern of MT creating more inequity in healthcare resources tends to follow egalitarianism theory for resource distribution that favours equality among populations by the removal of inequalities among people [57].

## Conclusion

More generally, most of the papers reviewed reveal conceptual vagueness of ethical issues associated with MT in Africa. Most of the work does not fully articulate the concerns, and tend not to draw from ethical theories and principles in a systematic way. Much of the evidence reviewed does not differentiate ethical issues from the other more nuanced ways of making choices, including religion and law. In identifying the ethical issues, most work reviewed is one-sided, failing to fully employ ethical theories and principles as helpful frameworks in more comprehensively addressing the identified issues. In not referring to ethical theories and principles in a comprehensive way, most of the literature fails to project issues at hand as ethical issues and/or justify why one chosen course of action/view is and should be preferred over the other. In particular, the lack of theory makes it difficult to grasp the very existence and nature of the identified issues, thereby making the analysis of empirical reality beneath the surface of MT in Africa elusive.

In conclusion, more empirical work is dearly needed to shed light on biomedical ethical issues associated with MT in Africa, including individual-level biomedical ethics of physician-assisted suicide, end-of-life decision-making, withdrawal of treatment and genetic testing. Population-level case-specific descriptions of experiences of individuals and populations with respect to distributive justice in healthcare is also needed.
